# Low adiponectin is associated with diastolic dysfunction in women: a cross-sectional study from the Tromsø Study

**DOI:** 10.1186/s12872-017-0509-2

**Published:** 2017-03-14

**Authors:** Jon V. Norvik, Henrik Schirmer, Kirsti Ytrehus, Trond G. Jenssen, Svetlana N. Zykova, Anne E. Eggen, Bjørn O. Eriksen, Marit D. Solbu

**Affiliations:** 1 Metabolic and Renal Research Group, UiT The Arctic University of Norway, N-9037 Tromsø, Norway; 2 Section of Nephrology, University Hospital of North Norway, N-9038 Tromsø, Norway; 3 Department of Cardiology, University Hospital of North Norway, N-9038 Tromsø, Norway; 4 Department of Transplant Medicine, Oslo University Hospital Rikshospitalet, N-0424 Oslo, Norway; 5 Department of Community Medicine, UiT The Arctic University of Norway, N-9037 Tromsø, Norway; 6 Cardiovascular Research Group IMB, UiT The Arctic University of Norway, N-9037 Tromsø, Norway; 7 Cardiovascular Research Group IKM, UiT The Arctic University of Norway, N-9037 Tromsø, Norway

**Keywords:** Diastolic dysfunction, Heart failure, HFpEF, Cardiovascular disease, Cardiac remodeling, Adiponectin, Women, Cross-sectional, Epidemiology

## Abstract

**Background:**

Heart failure with preserved ejection fraction is closely associated with diastolic dysfunction and related to obesity and female sex. We investigated whether adiponectin, an adipocyte-secreted protein hormone with cardioprotective effects, was associated with indices of diastolic dysfunction, and whether the association was sex dependent.

**Methods:**

We conducted a cross-sectional study on 1165 women and 896 men without diabetes. We stratified the multivariable adjusted logistic regression analyses and the fractional polynomial regression analyses according to sex, with echocardiographic markers of diastolic dysfunction as dependent variables, and adiponectin as the independent variable of interest.

**Results:**

Decreased adiponectin was associated with higher odds of average tissue Doppler e’ < 9 in women (odds ratio [OR] 1.17 per 1 μg/mL adiponectin decrease, 95% confidence interval [CI] 1.04–1.30), but not in men (p for interaction with sex 0.04). Women, but not men, had higher odds of E/e’ ratio ≥ 8 with lower adiponectin (OR 1.12 per 1 μg/mL adiponectin decrease, 95% CI 1.02–1.24, p for interaction with sex 0.04). Adiponectin in the lower sex-specific tertile was associated with increased odds of concentric left ventricular hypertrophy in women (OR 2.44, 95% CI 1.03–5.77), but with decreased odds in men (OR 0.32, 95% CI 0.11–0.88, p for interaction with sex 0.002), and decreased odds of eccentric hypertrophy in men only (OR 0.53, 95% CI 0.33–0.88, p for interaction with sex 0.02). Adiponectin in the lower sex-specific tertile was associated with moderately enlarged left atria in women only (OR 1.43, 95% CI 1.01–2.03, p for interaction with sex 0.04). Finally, adiponectin had a non-linear relationship with left ventricular mass in women only, with exponentially increasing left ventricular mass with lower adiponectin levels (p for interaction with sex 0.01).

**Conclusions:**

Low adiponectin was associated with higher odds of indices of diastolic dysfunction in women, but lower odds of indices of diastolic dysfunction in men. Lower adiponectin was associated with increased left ventricular mass in women only.

## Background

Diastolic dysfunction (DD) is a condition characterized by abnormal cardiac relaxation, stiffness or filling. Heart failure (HF) with preserved ejection fraction (HFpEF) is a clinical syndrome with symptoms and signs of HF, but with normal or only mildly reduced ejection fraction, and is closely associated with DD [1]. In developed countries, the prevalence of HF is about 1–2% of the adult population, and ≥ 10% among persons > 70 years of age [1]. About half of HF patients have HFpEF [2]. The EURObservational Research Program: the Heart Failure Pilot Survey (ESC_HF Pilot) conducted a prospective 1-year survey among 136 cardiology centers in 12 countries with over 5.000 HF patients enrolled [3]. The all-cause mortality rate after 1 year was 13.4% in acute HFpEF and 5.9% in chronic, stable HFpEF. DD in itself, not accompanied by HF, is associated with increased all-cause mortality, and it is often asymptomatic [4]. Despite HFpEF being a major and growing health problem in the USA and Europe, the understanding of this condition is still scarce and there is no medical treatment of proven benefit for HFpEF [5]. In particular, HFpEF is associated with female sex‚ increasing age and obesity [1]. The reasons for the female preponderance for HFpEF remain unsolved [6]. It is therefore of interest to examine features associated with DD, among them metabolic risk factors, for better understanding of possible sex-specific mechanisms leading to HFpEF.

Adipose tissue is an endocrine organ that produces a range of biologically active substances, such as the adipokines. They constitute a group of protein hormones with functions in the regulation of energy metabolism, insulin sensitivity, inflammation, atherosclerosis and cell proliferation [7]. Biologically important members include, among others, tumor necrosis factor-α, interleukin-6, interleukin-10, omentin, leptin and adiponectin [8]. The latter is abundantly produced and secreted by adipose tissue, and during the previous decade adiponectin has been shown to have antidiabetic, anti-inflammatory, anti-atherogenic and cardioprotective effects [7]. Low levels of adiponectin are in longitudinal studies associated with the development of insulin resistance [9] and hypertension [10], and adiponectin is negatively associated with body mass index (BMI) and body-fat [11]. Reduced plasma adiponectin is associated with the metabolic syndrome [12] and higher risk of myocardial infarction [13], although, paradoxically, in chronic HF adiponectin levels tend to be high, and increasing with disease severity [14]. It has been postulated that uric acid can affect adipocytes by inducing downregulation of adiponectin [15]. Thus, there may be a biologically relevant interplay between uric acid and adiponectin.

The relationship between adiponectin and DD has not been thoroughly investigated. Since there is a female preponderance for HFpEF, it is of relevance to assess whether these associations may differ by sex. Therefore, we wanted to do cross-sectional analyses and examine the associations between adiponectin and echocardiographic indices of DD in a large cohort of men and women from the general, non-diabetic population.

## Methods

### Study population

The Tromsø Study is a population-based, prospective study of various health issues and chronic diseases. It consists of seven surveys referred to as Tromsø 1-7, which have been conducted in the municipality of Tromsø, Norway, from 1974 to 2016 [16]. The population of the present study is the participants of the sixth wave of the Tromsø Study, conducted in 2007–2008 [17]. A total of 12,984 subjects (6930 women and 6054 men, 65.7% of the invited population) took part in the survey. All individuals who had attended the extended study of the fourth wave of the Tromsø Study, and were aged 50–62 years or 75–84 years plus a 20% random sample of persons aged 63–74 years (a total of 7206 subjects) were invited to an extended examination. Of these, 2243 persons were randomly selected for echocardiography. We excluded the subjects with prevalent diabetes at baseline (*n* = 182; defined as HbA1c ≥ 6.5%, non-fasting glucose ≥ 10.0 mmol/L, under anti-diabetic treatment or self-reported diabetes). This left 2061 subjects in the final cohort.

### Measurements

The subjects completed a questionnaire, which provided information on physical activity, prevalent diabetes, smoking habits and use of medication, as previously described [16, 17]. Use of blood pressure lowering drugs was assessed through self-report (questionnaire) based on the question “Do you use, or have you used blood pressure lowering drugs? Currently/Previously but not now/Never used”. Participants also reported drugs used on a regular basis the preceding four weeks; this was coded according to the Anatomical Therapeutic Chemical (ATC) classification system version 2007 (http://www.whocc.no/). We classified smoking habits as current smokers and non-smokers, and physical activity as active (≥ 1 h physical activity with prominent perspiration or breathlessness per week) or inactive (all others). We calculated BMI as weight (kg)/height (m)^2^. Blood pressure was recorded after a two minutes rest in 1-minute interval triplicate by an automated device (Dinamap ProCare Monitor 300, GE Healthcare, Norway). We used the mean of the second and the third measurement. Blood samples were non-fasting. Total adiponectin was analyzed by an ELISA method (DY1065 kit from R&D Systems, Inc., Minneapolis, MN) in serum that had been stored at -70°. Uric acid, serum total cholesterol, cystatin C, triglycerides, HbA1c and serum creatinine were analyzed as previously described [17, 18]. Urine was collected and analyzed as previously described [18], and the median value of three albumin-creatinine ratio values was used, calculated from urine albumin and creatinine of first-void morning urine samples taken on three consecutive days. We calculated the estimated glomerular filtration rate (eGFR) according to the CKD-EPI creatinine-cystatin C equation [19].

### Echocardiography

Transthoracic Echocardiography was performed with an Acuson Sequoia C512 Ultrasound System (Siemens Medical Solutions. Mountain View, California, USA) with a combined 3.5 MHz second harmonic ultrasound and 2.5 MHz Doppler probe. M-mode, 2D and Doppler echocardiographic recordings were obtained and analyzed for all participants. A sonographer was the primary performer of the echocardiography examinations, with an experienced cardiologist as backup and supervisor. Measurements were done online by the sonographer as the recording was done. The measurements were also done online for the intraobserver and interobserver study where the reproducibility of measurements was assessed by new recordings as basis for comparison. Mean intraobserver variability ± standard deviation (SD) for the sonographer for E/A ratio was 0.007 ± 0.28, for left atrial (LA) diameter 0.01 ± 0.49 cm, and for E-wave deceleration time (EDT) 6 ± 53 ms. The mean interobserver variability ± SD between the sonographer and the cardiologist was 0.015 ± 0.29 for E/A ratio, 0.04 ± 0.38 cm for LA diameter, and 5 ± 38 ms for EDT.

Left ventricular (LV) mass was calculated by the cube formula at end-diastole (LV mass = 0.8 x [1.04 x {interventricular septum thickness + LV internal diameter + posterior wall thickness}^3^ – {LV internal diameter}^3^] + 0.6 g) [20]. Relative wall thickness (RWT) was calculated as (relative wall thickness = [2 x posterior wall thickness]/LV internal diameter at end-diastole) [20]. We indexed LA size and LV mass by body surface area calculated by the Du Bois formula (body surface area = [weight {kg} ^0.425^ × height {cm} ^0.725^] × 0.007184).

### Diastolic dysfunction

We used measurements of LA size, LV remodeling, tissue Doppler and Doppler of mitral flow as parameters of DD, and the cut-offs were set according to previously published data and international guidelines [1, 20–22]. We defined LA size as normal (< 2.2 cm/m^2^), moderately enlarged (2.2–2.79 cm/m^2^) and severely enlarged (≥ 2.8 cm/m^2^). Isovolumetric relaxation time (IVRT) was either reduced (< 60 ms), normal (60–110 ms), or prolonged (> 110 ms). LV hypertrophy was defined as LV mass > 95 g/m^2^ in women and > 115 g/m^2^ in men. LV remodeling was defined as normal, concentric remodeling (no hypertrophy and RWT > 0.42), concentric hypertrophy (hypertrophy and RWT > 0.42), and eccentric remodeling (hypertrophy and RWT ≤ 0.42). The early myocardial peak velocity of the mitral annulus, tissue Doppler e’ wave (the average of the septal e’ and lateral e’ measurements), was defined as decreased (< 9 cm/s) or normal (≥ 9 cm/s). E/e’, the ratio of peak early LV filling (E-wave) and average tissue Doppler e’ wave, was stratified into normal (< 8) and increased (≥ 8). E/A ratio, the ratio of the E-wave and peak late LV filling (A-wave), was divided into low (< 1.0), normal (1.0–2.0) and high (> 2.0). We defined EDT, the deceleration time of early filling velocity, into low (< 140 ms), normal (140–220 ms) and high (> 220 ms).

### Statistics

The population characteristics are given as number and percentage of total or mean ± SD, except for albumin-creatinine ratio, which is given as median and interquartile range. Independent sample t-tests and chi square tests were applied to compare baseline variables between women and men. For each of the indices of DD, we tested the associations with the continuous variable of adiponectin or adiponectin divided into the lowest sex-specific tertile vs. all others, and the covariates sex, age, waist circumference, mean systolic blood pressure, mean diastolic blood pressure, physical activity, smoking, use of blood pressure lowering drugs, total cholesterol, HDL cholesterol, triglycerides, eGFR, and albumin-creatinine ratio. In analyses with dichotomized indices of DD, we employed multivariable binary logistic regression models, and in analyses assessing markers of DD classified into more than two categories (i.e. E/A ratio, EDT, IVRT, LV remodeling and LA size) we used multivariable multinomial logistic regression models, with the normal categories as references. The indices of DD were the dependent variables in these analyses, and adiponectin was the independent variable of interest. We also did the analyses with a combined variable for DD: average e’ wave < 9 plus LA size ≥ 2.2 cm/m^2^, an approximation of a definition of DD given by guidelines [23]. We tested for interaction between sex and adiponectin for the association with each echocardiographic marker of DD using two-way cross products between sex and the continuous variable of adiponectin or the indicator variable of adiponectin divided into lowest sex-specific tertile vs. all others. With the same covariates as listed above, we examined the association of adiponectin with the two continuous variables of cardiac remodeling, LV mass and LA size, with linear regression analyses. We tested for non-linearity by applying fractional polynomial models, which estimated the best prediction of the outcome variable from the power transformations -2, -1, - 0.5, 0 (log transformation), 0.5, 1, 2, 3, on the same linear regression models. We tested for interaction between sex and adiponectin for the association with the continuous variables using two-way cross products between sex and adiponectin as a continuous variable. A two-sided *p*-value of < 0.05 was considered significant. We performed all the analyses with SPSS software (IBM Corp. Released 2015. IBM SPSS Statistics for Windows, Version 23.0. Armonk, NY: IBM Corp), except for the polynomial fractional analyses, which were done with Stata software (StataCorp. 2015. Stata Statistical Software: Release 14. College Station, TX: StataCorp LP).

## Results

### Population characteristics

The indices of DD and cardiac remodeling examined in the present study are listed in Table 1, along with the number of men and women with each echocardiographic marker of DD.

**Table 1:** Indices of diastolic dysfunction and LV remodeling

Index	Normal values	Diastolic dysfunction and LV remodeling	Women with index at baseline, n (%)	Men with index at baseline, n (%)
E/A ratio	1.0–2.0	< 1.0; > 2.0	< 1.0: *n* = 629 (54.0%); > 2.0: *n* = 12 (1.0%)	< 1.0: *n* = 505 (58.8%); > 2.0: *n* = 14 (1.6%)
EDT	140–220 ms	< 140 ms; > 220 ms	< 140 ms: *n* = 120 (10.3%); > 220 ms; *n* = 163 (14.0%)	< 140 ms: *n* = 73 (8.1%); > 220 ms; *n* = 188 (21.0%)
IVRT	60–110 ms	< 60 ms; > 110 ms	< 60 ms: *n* = 25 (2.1%); > 110 ms: *n* = 23 (2.0%)	< 60 ms: *n* = 12 (1.3%); > 110 ms: *n* = 29 (3.2%)
Average e’ wave	≥ 9 cm/s	< 9 cm/s	*n* = 184 (15.8%)	*n* = 125 (14.0%)
E/e’ ratio	< 8	≥ 8	*n* = 247 (21.2%)	*n* = 149 (16.6%)
LA size	< 2.2 cm/m^2^	Moderately enlarged: 2.2–2.79 cm/m^2^; severely enlarged: ≥ 2.8 cm/m^2^	Moderately enlarged: *n* = 319 (27.4%); severely enlarged: *n* = 26 (2.2%)	Moderately enlarged: *n* = 176 (19.6%); severely enlarged: *n* = 14 (1.6%)
LV remodeling	LV mass ≤ 95 g/m^2^ in women or ≤ 115 g/m^2^ in men, and RWT ≤ 0.42	Concentric remodeling: LV mass ≤ 95 g/m^2^ in women or ≤ 115 g/m^2^ in men, and RWT > 0.42; concentric hypertrophy: LV mass > 95 g/m^2^ in women or > 115 g/m^2^ in men, and RWT > 0.42; eccentric hypertrophy: LV mass > 95 g/m^2^ in women or > 115 g/m^2^ in men, and RWT ≤ 0.42	Concentric remodeling: *n* = 45 (3.9%); concentric hypertrophy: *n* = 40 (3.4%); eccentric hypertrophy: *n* = 206 (17.7%)	Concentric remodeling: *n* = 39 (4.4%); concentric hypertrophy: *n* = 35 (3.9%); eccentric hypertrophy: *n* = 147 (16.4%)

*Abbreviations*: *EDT* E-wave deceleration time, *IVRT* Isovolumetric relaxation time, *LA* Left atrium, *LV* Left ventricle, *RWT* Relative wall thickness

The population characteristics, specified for sex, are given in Table 2. Most biometrics, including age, differed significantly between the sexes. This included the echocardiography parameters, except for E/A ratio. Of the non-echocardiographic variables, only systolic blood pressure, eGFR, albumin-creatinine ratio, daily smokers, and use of blood pressure lowering drugs did not significantly differ between the sexes.

**Table 2:** Baseline characteristics of cohort divided by sex (*n* = 2061)

	Women (*n* = 1165)		Men (*n* = 896)		P between sex
Age, years	64.2	±11.7	65.6	±10.4	0.004
Adiponectin, μg/mL	4.64	±2.07	3.16	±1.48	<0.001
Uric acid, μmol/L	280.2	±66.3	349.1	±71.5	<0.001
Systolic blood pressure, mm Hg	141.7	±26.2	143.5	±20.6	0.094
Diastolic blood pressure, mm Hg	76.5	±10.3	81.7	±10.3	<0.001
BMI, kg/m^2^	26.4	±4.3	27.2	±3.6	<0.001
Waist circumference, cm	91.0	±11.3	100.1	±10.2	<0.001
Total cholesterol, mmol/L	5.89	±1.07	5.57	±1.11	<0.001
Serum HDL, mmol/L	1.72	±0.43	1.42	±0.40	<0.001
Triglycerides, mmol/L	1.37	±0.70	1.53	±0.88	<0.001
eGFR, mL/min/1.73 m^2^	89.6	±17.9	86.6	±14.5	0.187
Albumin-creatinine ratio, mg/mmol	0.36	[0.21–0.73]	0.37	[0.17–0.87]	0.798
Daily smoking, n	210	18.0%	141	15.7%	0.171
Physically active, n	316	27.1%	285	31.8%	0.020
Use of blood pressure lowering drugs, n	383	32.9%	312	34.8%	0.354
Left ventricular mass, g/m^2^	84.1	±21.5	101.0	±26.7	<0.001
Left atrial size, cm/m^2^	2.09	±0.30	2.02	±0.28	<0.001
E-wave deceleration time, ms	182.5	±43.8	193.7	±51.2	<0.001
E/A ratio	1.02	±0.34	0.99	±0.35	0.080
Left ventricular ejection fraction, %	72.1	±7.8	68.7	±8.8	0.033
Left ventricular ejection fraction < 50%, n	7	0.6%	14	1.6%	0.032
Average e', cm/s	11.28	±2.58	10.97	±2.24	0.005
E/e' ratio	6.88	±2.09	6.50	±1.98	<0.001

*T*-test or chi square were used to compare values between men and women

The Tromsø Study

Values are given as mean (SD), median (IQR), or number (%)

*Abbreviations*: *BMI* Body mass index, *eGFR* estimated glomerular filtration rate, *HDL* High-density lipoprotein

### Multivariable regression models

Multivariable logistic regressions with the indices of DD that were significantly associated with adiponectin (i.e., average e’ < 9, LA size ≥ 2.8 cm/m^2^) as the dependent variables are shown in Table 3. Decreased adiponectin was associated with significantly higher odds of having average e’ < 9. On the other hand, lower adiponectin was associated with reduced odds of severely enlarged LA size. There were no significant associations between continuous adiponectin and the indicator variables for E/A ratio, E/e’ ratio, EDT, IVRT, or LV remodeling, nor for moderately enlarged atria (not shown).

**Table 3:** Multivariable logistic regression models with some indices of diastolic dysfunction as dependent variables

	Average e' < 9			LA size ≥ 2.8 cm/m^2a^		
	Odds ratio	95% CI	*P* value	Odds ratio	95% CI	*P* value
Adiponectin (per 1 μg/mL decrease)	1.12	[1.02–1.22]	0.015	0.78	[0.66–0.93]	0.004
Uric acid (per 59 μmol/L increase)	1.08	[0.94–1.25]	0.282	1.68	[1.21–2.32]	0.002
Sex (male vs. female)	0.59	[0.41–0.86]	0.005	0.54	[0.19–1.49]	0.233
Age (per 5 year increase)	1.76	[1.56–1.99]	<0.001	2.52	[1.76–3.57]	<0.001
Waist circumference (per 5 cm increase)	1.00	[0.93–1.08]	0.971	0.77	[0.91–0.99]	0.009
Mean systolic blood pressure (per 5 mm Hg increase)	1.05	[1.01–1.09]	0.025	1.01	[0.91–1.11]	0.888
Mean diastolic blood pressure (per 5 mm Hg increase)	1.15	[1.05–1.25]	0.002	0.95	[0.77–1.16]	0.584
Albumin-creatinine ratio (per 1 mg/mmol increase)	1.01	[0.99–1.02]	0.334	1.04	[1.01–1.06]	0.007
Total cholesterol (per 1 mmol/L increase)	0.90	[0.77–1.04]	0.142	1.10	[0.75–1.62]	0.615
HDL cholesterol (per 1 mmol/L increase)	1.17	[0.75–1.82]	0.497	0.30	[0.09–1.04]	0.057
Triglycerides (per 1 mmol/L increase)	1.19	[0.94–1.51]	0.146	0.67	[0.29–1.56]	0.352
eGFR (per 5 mL/min/1.73 m^2^ increase)	1.02	[0.96–1.08]	0.609	1.17	[1.01–1.36]	0.040
Daily smoking (yes vs. no)	1.47	[0.99–2.19]	0.059	1.72	[0.59–5.01]	0.318
Physically active (yes vs. no)	1.08	[0.76–1.53]	0.672	1.42	[0.51–3.90]	0.500
Use of blood pressure lowering drugs (yes vs. no)	1.26	[0.92–1.71]	0.148	2.17	[0.95–4.93]	0.065

The Tromsø Study

Covariates: Age, sex, waist circumference, mean systolic blood pressure, mean diastolic blood pressure, use of blood pressure lowering drugs, total cholesterol, HDL cholesterol, triglycerides, eGFR, albumin-creatinine ratio, physically active, daily smoker and uric acid

*Abbreviations*: *eGFR* estimated glomerular filtration rate, *HDL* high-density lipoprotein, *LA* left atrium

^a^Compared to reference category, LA size < 2.2 cm/m^2^

**Fig. 1:**
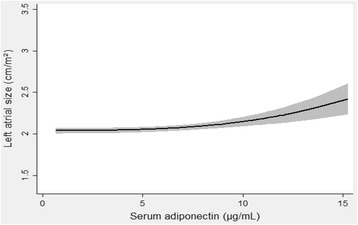
Multivariable fractional polynomial regression with left atrial size as the dependent variable, and adiponectin as the independent variable of interest. Shaded regions denote 95% confidence interval. Covariates: Age, sex, waist circumference, mean systolic blood pressure, mean diastolic blood pressure, use of blood pressure lowering drugs, total cholesterol, high-density lipoprotein, triglycerides, estimated glomerular filtration rate, physically active, daily smoker, albumin-creatinine ratio and uric acid

Table 4 displays the continuous variables for LV mass and LA size as dependent, and adiponectin as the independent variable of interest in multivariable linear regression models. Adiponectin was significantly and positively associated with LA size. Figure 1 shows the result of multivariable fractional polynomial regression and the non-linear (fractional power 3, *p* < 0.001) relationship between adiponectin and LA size, which had a better fit than the linear model. Uric acid did not interact significantly with adiponectin for any of the analyses.

**Table 4:** Linear regression models with parameters of cardiac remodeling as dependent variables

	Adiponectin (per 1 μg/mL increase)			
	β	95% CI	t	*P* value
Left ventricular mass (g/m^2^)	−0.59	−1.29 to 0.11	−1.66	0.098
Left atrial size (cm/m^2^)	0.013	0.01 to 0.02	3.29	0.001

The Tromsø Study

Covariates: Age, sex, waist circumference, mean systolic blood pressure, mean diastolic blood pressure, use of blood pressure lowering drugs, total cholesterol, high-density lipoprotein cholesterol, triglycerides, estimated glomerular filtration rate, albumin-creatinine ratio, physically active, daily smoker and uric acid

### Sex differences

**Fig. 2:**
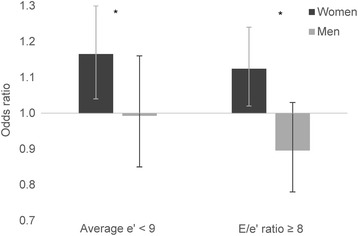
Multivariable binary logistic regression models divided by sex with average e’ < 9 and E/e’ ratio ≥ 8 as dependent variables. Odds ratio are per 1 μg/mL adiponectin decrease. Whiskers represent 95% confidence interval. * = p for interaction with sex < 0.05. Covariates: Age, waist circumference, mean systolic blood pressure, mean diastolic blood pressure, use of blood pressure lowering drugs, total cholesterol, high-density lipoprotein, triglycerides, estimated glomerular filtration rate, physically active, daily smoker, albumin-creatinine ratio and uric acid

Significant interaction between adiponectin (continuous variable) and sex was found for the association with two markers of DD, average e’ < 9 and E/e’ ratio ≥ 8. Sex stratified logistic regression analyses for these markers are illustrated in Fig. 2. Lower adiponectin was associated with higher odds of average e’ < 9 in women (odds ratio [OR] 1.17 per 1 μg/mL adiponectin decrease, 95% confidence interval [CI] 1.04–1.30, *p* = 0.01), but not men (OR 0.99, 95% CI 0.85–1.16, *p* = 0.93), p for interaction between adiponectin and sex was 0.04. Lower adiponectin was associated with higher odds of E/e’ ratio ≥ 8 in women (OR 1.12 per 1 μg/mL adiponectin decrease, 95% CI 1.02–1.24, *P* = 0.02), but not in men (OR 0.90, 95% CI 0.78–1.03, *p* = 0.13, p for interaction between adiponectin and sex 0.04).

**Fig. 3:**
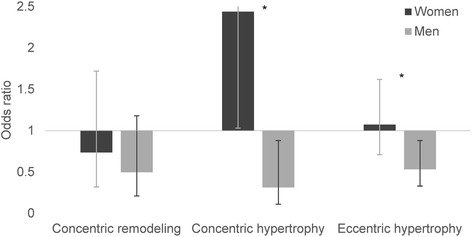
Multivariable multinomial logistic regression models divided by sex with left ventricular remodeling as the dependent variable. Each category of left ventricular remodeling displayed is compared to the normal category. Odds ratios are for the individuals in the sex-specific lower adiponectin tertile compared to the two upper tertiles. Whiskers represent 95% confidence interval. * = p for interaction with sex < 0.05. Covariates: Age, waist circumference, mean systolic blood pressure, mean diastolic blood pressure, use of blood pressure lowering drugs, total cholesterol, high-density lipoprotein, triglycerides, estimated glomerular filtration rate, physically active, daily smoker, albumin-creatinine ratio and uric acid

**Fig. 4:**
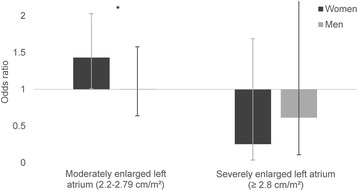
Multivariable multinomial logistic regression models divided by sex with left atrial size as the dependent variable. Each category of left atrial size displayed is compared to normal category, left atrial size < 2.2 cm/m^2^. Odds ratios are for the individuals in the sex-specific lower adiponectin tertile compared to the two upper tertiles. Whiskers represent 95% confidence interval. * = p for interaction with sex < 0.05. Covariates: Age, waist circumference, mean systolic blood pressure, mean diastolic blood pressure, use of blood pressure lowering drugs, total cholesterol, high-density lipoprotein, triglycerides, estimated glomerular filtration rate, physically active, daily smoker, albumin-creatinine ratio and uric acid

Figures 3 and 4 display the indices of DD for which sex and the categorized adiponectin variable interacted significantly, namely LV remodeling and LA size. Adiponectin values in the lower tertile was associated with increased odds of concentric LV hypertrophy (OR 2.44, 95% CI 1.03–5.77, *p* = 0.04) in women, but in men reduced odds for the same outcome (OR 0.32, 95% CI 0.11–0.88, *p* = 0.03, p for interaction with sex 0.002), compared to normal LV architecture, as shown in Fig. 3. Moreover, adiponectin in the lower tertile was associated with lower odds of eccentric LV hypertrophy in men (OR 0.53, 95% CI 0.33–0.88, *p* = 0.01), but not women (OR 1.07, 95% CI 0.71–1.62, *p* = 0.74 p for interaction 0.02).

Figure 4 illustrates the ORs of the association between adiponectin and LA size divided by sex. Compared to normal LA size, adiponectin in the lower tertile was associated with moderately enlarged LA in women (OR 1.43, 95% CI 1.01–2.03, *p* = 0.04), but not in men (OR 1.01, 95% CI 0.64–1.58, *p* = 0.98, p for interaction with sex 0.04). There was no significant association between severely enlarged LA and adiponectin for either sex.

**Fig. 5:**
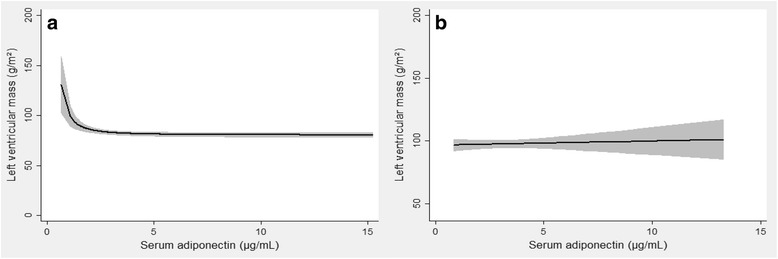
Multivariable fractional polynomial regression with left ventricular mass as the dependent variable, and adiponectin as the independent variable of interest with cohort divided into women (**a**) and men (**b**). Shaded regions denote 95% confidence interval. Covariates: Age, waist circumference, mean systolic blood pressure, mean diastolic blood pressure, use of blood pressure lowering drugs, total cholesterol, high-density lipoprotein, triglycerides, estimated glomerular filtration rate, physically active, daily smoker, albumin-creatinine ratio and uric acid

Multivariable fractional polynomial regression revealed that lower adiponectin was associated with increased LV mass in women (fractional power -2, *p* = 0.001), but not men (*p* = 0.66, p for interaction with sex 0.01), as displayed in Fig. 5a and b, respectively.

For a combination variable of DD consisting of subjects with both LA size ≥ 2.2 cm/m^2^ and average e’ < 9, the indicator variable of low adiponectin (lower sex-specific tertile vs. all others) interacted with sex (*p* = 0.03). However, adiponectin did not significantly predict this outcome for neither women (OR 1.39, 95% CI 0.98–1.97, *p* = 0.07) nor men (OR 0.95, 95% CI 0.61–1.50, *p* = 0.83).

## Discussion

To our knowledge, this is the first study to demonstrate sex differences in the association between adiponectin and DD in a general, non-diabetic population. In this study of 1165 women and 896 men, we have shown that a lower adiponectin was associated with higher odds of indices of DD in women, including e’ < 9, E/e’ ratio ≥ 8, concentric LV hypertrophy, and moderately enlarged LA. In addition, lower adiponectin was associated with higher LV mass in women. These associations were not found in men. Rather, low adiponectin was associated with decreased odds of concentric LV hypertrophy and eccentric LV hypertrophy in men. Except for a severely enlarged atrium, we found no marker of DD that was associated with a high adiponectin in women, and conversely, we found no marker of DD at all that was associated with a low adiponectin in men.

Traditionally, high adiponectin levels have been regarded as favorable in terms of cardiac health, but research has challenged this view, and the role and action of adiponectin in heart disease is not clear. In general, low adiponectin has been associated with the development of coronary heart disease in healthy subjects [24], but high adiponectin was a risk factor for severity of HF and mortality in patients with chronic HF [25]. A current theory to this equivocal relationship of adiponectin and heart disease is that high levels are beneficent in healthy subjects, but that adiponectin levels can be upgraded as a compensatory mechanism in the face of chronic HF [14]. This may explain, in our study, why in women low adiponectin was associated with moderately enlarged LA, but higher adiponectin was associated with severely enlarged LA. Adiponectin could also increase simply due to reduced kidney function, which commonly coexists with HF [26].

To our knowledge, only a few studies have examined sex differences in the association between adiponectin and DD. In one paper, a cohort consisting of 193 patients undergoing cardiac catheterization for coronary artery disease was divided by sex. The authors found a negative relationship between adiponectin and DD (evaluated by correlation with LV end-diastolic pressure, and Tau, the time constant of the decrease in LV pressure), but they could not report a significant sex difference in this association [27]. A recent population based study of 556 individuals did not find any association between DD and adiponectin for either sex [28]. The study categorized patients into DD grades: grade I (mild DD) if lateral e’ < 10 cm/s, E/A ratio < 0.8, EDT > 200 ms, and E/e’ ratio ≤ 8; grade II (moderate DD) if lateral e’ < 10 cm/s, E/A ratio 0.8–1.5, EDT 160–200 ms, and E/e’ ratio 9–12; and grade III (severe DD), if lateral e’ < 10 cm/s, E/A ratio ≥ 2, EDT < 160 ms, and E/e’ ratio ≥ 13. The fact that our population was much larger and used somewhat different DD indices, may account for the divergent results.

Experimental studies may shed some light on the effects of adiponectin on the development of DD and the sex differences in the overall action of adiponectin. In adiponectin deficient mice, pressure overload resulted in concentric cardiac hypertrophy and mortality to a greater degree than in pressure overloaded wild type [29]. Another study in adiponectin deficient mice, in which the animals were subjected to aldosterone-induced hypertension in addition to uninephrectomy, showed that hypoadiponectinaemia exacerbated hypertension-induced DD with increased LV mass, increased E/A ratio, reduced e’ wave and increased E/e’ ratio in the adiponectin deficient mice compared to the wild type [30]. Transgenic mice that overexpressed adiponectin were protected from DD after pressure overload compared to wild type [31]. Induced activity of the enzyme heme oxygenase-1 (HO-1) has been associated with reduced oxidative stress, increased insulin sensitivity and increased adiponectin levels [32, 33]. One study pharmacologically induced HO-1 in both lean and obese female and male mice, and discovered that although female obese mice had higher levels of several inflammatory cytokines than male obese mice, HO-1 induction and a subsequent increase in adiponectin, reduced inflammatory cytokines to similar levels in females and males [34]. Similar results were found in another study on obese female and male mice that administered a peptide that increased HO-1 and adiponectin levels [35], and the effects were independent of body weight in the female animals only. In both these studies, adiponectin levels were similar in obese male and female mice, but treatment raised adiponectin levels significantly higher in females than males. These animal studies are suggestive of sex differences in concentration/production and activity of adiponectin, especially in the obese subjects. Indeed, adiponectin levels are generally higher in women than in men, perhaps in part because of a negative correlation with testosterone [36], and post-menopausal women have lower levels of adiponectin [37]. Also in humans, the relative sex difference in adiponectin is less pronounced in obese than non-obese [38]. Additionally, one study found DD to be more strongly correlated with abdominal adiposity in women than in men [24]. Although several studies have confirmed a positive correlation between insulin sensitivity and adiponectin in both sexes [38], one study found a lower number of adiponectin receptors in female skeletal muscle and speculates that this may result in a reduced insulin sensitizing effect of adiponectin in women compared with men [39]. Adiponectin can be separated into three complexes: low molecular weight form, middle molecular weight form, and high molecular weight form (HMW), and some suggest that the ratio of HWM to total adiponectin levels is more closely associated with insulin sensitivity than total levels alone [40]. HMW adiponectin was lower in men, but not in women, with diabetes and coronary artery disease [41]. The dissimilarity in adiponectin levels between men and women may be due solely to a higher level of HWM in women than men [40]. Unfortunately, only total adiponectin was available in our survey, so it was not possible for us to examine the HMW fraction in relation to DD and sex. In sum, it is possible that discrepancies between men and women in adipose tissue activity, distribution of adiponectin receptors and HMW adiponectin content have contributed to the sex differences in the association between adiponectin and DD reported in our study. With the above in mind, the facts that our cohort had a mean BMI > 25 kg/m^2^, i.e. within the overweight range, and a mean age in the mid-sixties may have influenced our results. The relationship between adiponectin, obesity, sex, and organ damage is a complex one and further studies are clearly needed.

The main strengths of this study were the large cohort size, the high attendance rate and the ability to correct for several covariates, including eGFR and use of antihypertensive medication, and the use of the newest guideline based definitions of DD. The most important limitation was its cross-sectional design. Other relevant limitations include the lack of fasting blood samples, and the single measurement of total adiponectin available. A quantification of HMW adiponectin could have provided more data in our models. Moreover, our estimation of LA was based on the diameter; LA volume measurements may have yielded additional information. Data on tricuspid regurgitation velocity would also have strengthened the study. Our exclusion of subjects with diabetes may be a further limitation. The fact that our cohort largely consisted of middle-aged, healthy Northern Europeans limits the study’s generalizability to other ethnic groups.

## Conclusions

Low adiponectin was associated with several indices of DD and increased LV mass in women, but not in men. Whether these associations are clinically relevant and could influence prediction and treatment of DD, should be further explored.

## Abbreviations

BMI: Body mass index
CI: Confidence interval
DD: Diastolic dysfunction
E/A ratio: Ratio of peak early LV filling (E-wave) and peak late LV filling (A-wave)
E/e’ ratio: Ratio of E-wave and e’ wave
e’: Early myocardial peak velocity of the mitral annulus
EDT: E-wave deceleration time
eGFR: Estimated glomerular filtration rate
HDL: High-density lipoprotein cholesterol
HF: Heart failure
HFpEF: Heart failure with preserved ejection fraction
HMW: High molecular weight
HO-1: Heme oxygenase-1
IVRT: Isovolumetric relaxation time
LA: Left atrium
LV: Left ventricle
OR: Odds ratio
RWT: Relative wall thickness
